# The central mechanism of acupuncture treatment with a long-lasting effect for functional dyspepsia: study protocol for a randomized controlled trial

**DOI:** 10.1186/s13063-018-2742-0

**Published:** 2018-07-13

**Authors:** Ruirui Sun, Xiaojuan Hong, Jing Guo, Shuai Yin, Peiming Feng, Lei Lan, Du Lei, Xiaoyan Liu, Xueling Suo, Tao Yin, Tingting Zhang, Liuyang Huang, Feifei Gao, Qiyong Gong, Fanrong Liang, Fang Zeng

**Affiliations:** 1 Acupuncture and Tuina School/The 3rd Teaching Hospital, Chengdu University of Traditional Chinese Medicine, 37# Shierqiao Road, Chengdu, 610075 Sichuan China; 2 First Affiliated Hospital of Henan University of Traditional Chinese Medicine, Zhengzhou, Henan Province China; 3 The 1st Teaching Hospital of Chengdu University of Traditional Chinese Medicine, Chengdu, Sichuan China; 4 Huaxi MR Research Center (HMRRC), Departments of Radiology, West China Hospital of Sichuan University, 37# Guo Xue Xiang, Chengdu, 610041 Sichuan China

**Keywords:** Acupuncture, Long-lasting effect, *Deqi*, Functional dyspepsia, Functional magnetic resonance imaging, Central mechanism, Clinical trial, Protocol

## Abstract

**Background:**

The mechanism of the long-lasting effect and the relationship between the long-lasting effect and the *deqi* sensation, which is the key of achieving the acupuncture effect, has not been well investigated. This trial focuses on investigating the possible central mechanism of the long-lasting effect influenced by the *deqi* sensation.

**Methods:**

A randomized controlled functional brain imaging trial is currently being conducted in Sichuan, China. In total 105 functional dyspepsia (FD) patients will be allocated into three groups: an acupuncture with *deqi* group, an acupuncture without *deqi* group, and a wait-list group. This trial will include a 2-week baseline period, a 4-week treatment period, and a 4-week follow-up period. During the 4-week treatment, patients in two acupuncture groups will receive 20 sessions of acupuncture treatment with or without *deqi*. The Nepean Dyspepsia Index (NDI) and the short form Leeds Dyspepsia Questionnaire (SF-LDQ) will be used to evaluate the clinical efficacy of acupuncture treatment at baseline, the end of treatment, and the end of the follow-up. Functional magnetic resonance imaging (fMRI) scans will be performed to detect cerebral functional changes in 25 patients in each group at three time points mentioned above. The clinical data and fMRI data will be analyzed, respectively. Correlation analysis will be conducted to investigate the relationship between cerebral functional changes and symptom improvement.

**Discussion:**

The results of this trial will allow us to compare the changes of acupuncture therapeutic effect at three time points (the baseline vs. the end of treatment vs. the end of follow-up), and investigate the potential central mechanism of the long-lasting effect influenced by acupuncture with *deqi*. This trial aims to re-identify the long-lasting effect of acupuncture and investigate its central mechanism, and to further explore the central influence of *deqi* sensation on the long-lasting effect.

**Trial registration:**

Chinese Clinical Trial Registry, IDF: ChiCTR-IOR-15006523. Registered on 5 June 2015.

**Electronic supplementary material:**

The online version of this article (10.1186/s13063-018-2742-0) contains supplementary material, which is available to authorized users.

## Background

The long-lasting effect of a treatment, also called the “long-term effect,” is the perception of a curative effect for a period of time after treatment cessation. It has been widely found following observation after medical therapy [1–3]. For acupuncture treatment, the long-lasting effect is considered as one of the most important features in its therapeutic effect, which has been recorded in classical ancient Chinese books and proved by multiple modern clinical trials [4–10]. The long-lasting effect has been reported in trials of acupuncture for treating migraine [10], chronic headache [5], primary insomnia [8], tinnitus [9], etc. Our previous study found that, compared with sham acupuncture, verum acupuncture may be associated with long-term reduction in migraine recurrence [10]. Furthermore, this long-lasting effect is considered to be closely related to the *deqi* sensation, the key of achieving acupuncture efficacy [11]. In one of our previous randomized clinical trials (RCTs) we found that acupuncture on real acupoints presented persistent effect in the follow-up compared with acupuncture on sham acupoints in patients with functional dyspepsia [4]. However, the central mechanism of the long-lasting effect of acupuncture remains unknown, and its influencing factors have not been well studied.

Functional dyspepsia (FD) is defined as the presence of early satiation, postprandial fullness, epigastric pain, or epigastric burning in the absence of an organic, systemic, or metabolic disease that could explain the symptoms according to the Rome III consensus [12]. It is one of the most common categories in functional gastrointestinal disorders (FGID). In Sweden, the prevalence of FD is between 11 and 20% [13]. In Asia, it ranges from 8 to 23% [14]. Although FD is not life-threatening, it significantly influences quality of life (QOL) [15] and leads to high medical costs [16]. Nowadays, many approaches, such as daily proton pump inhibitors (PPI), tricyclic antidepressants, anti-nociceptive agents, and prokinetic agent have been used to treat FD [17–21], but the therapeutic effect is unsatisfactory due to its multi-factor pathology. As a result, more and more patients tends to seek complementary and alternative methods.

We design a randomized controlled functional brain imaging trial with a 10-week observation period, aiming at: (1) re-identifying the long-lasting effect of acupuncture treatment for FD by comparing the changes of therapeutic effect at three time points (the baseline vs. the end of treatment vs. the end of follow-up); (2) investigating the central mechanism of the long-lasting effect of acupuncture; and (3) exploring the influence of the *deqi* sensation on the long-lasting effect and its potential central mechanism.

## Methods and design

### Study design

**Fig. 1:**
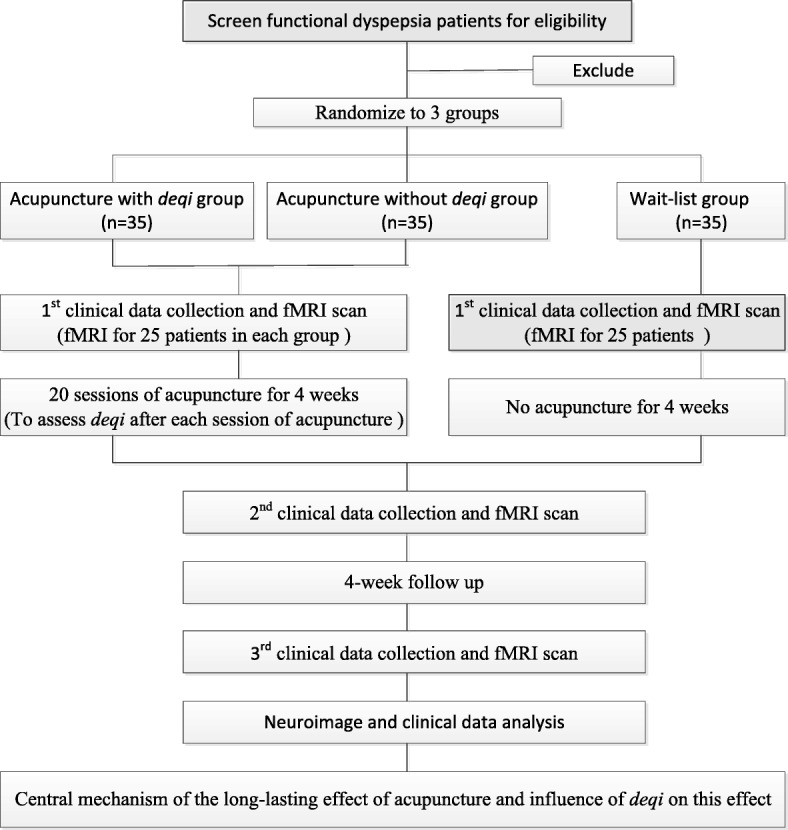
Study schedule for data collection. The informed consent and examination will be conducted after recruitment. Matched functional dyspepsia (FD) patients will be then randomized into three groups, where two acupuncture groups will receive treatment. Both clinical outcomes and functional magnetic resonance imaging (fMRI) scans will be performed at three time points including: the baseline, the end of acupuncture treatments, and the end of follow-up. Adverse events will be recorded in the case report at any time during the study. a. *NDI* Nepean Dyspepsia Index, *LDQ* Leeds Dyspepsia Questionnaire, *SAS* Self-Rating Anxiety Scale, *SDS* Self-Rating Depression Scale, *BAI* Beck Anxiety Inventory, *BDI* Beck Depression Inventory, *VAS* Visual Analog Scale

**Fig. 2:**
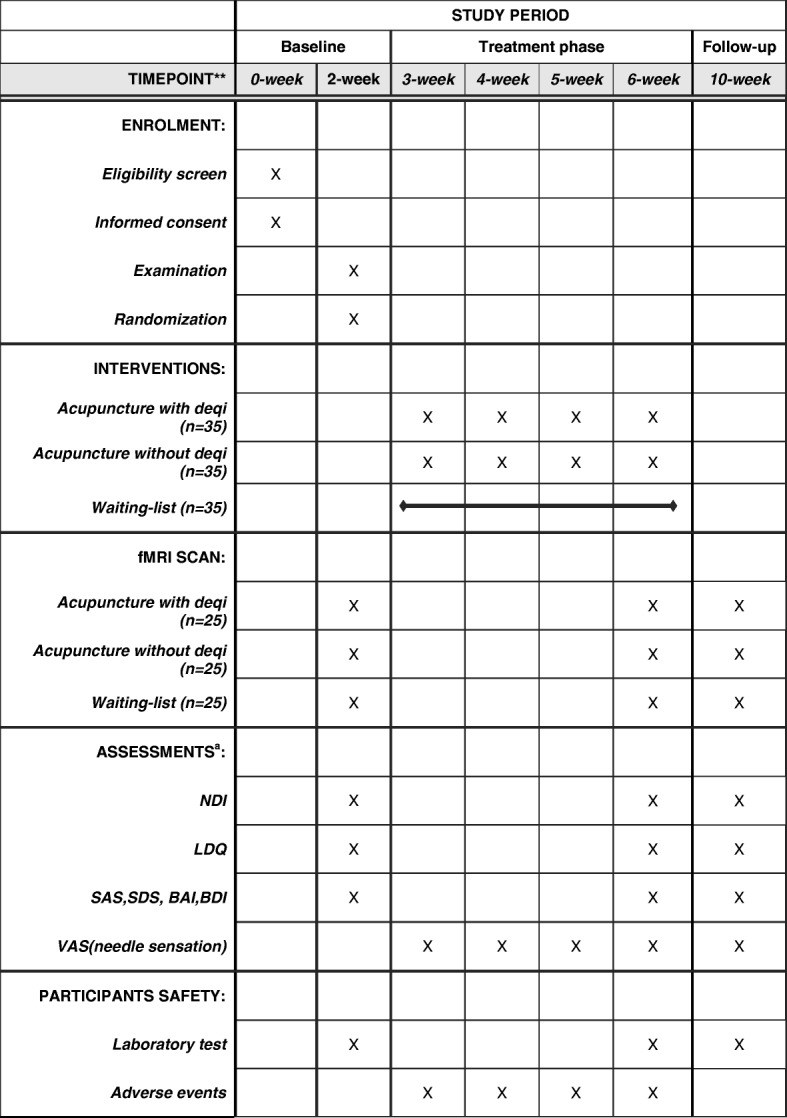
Standard Protocol Items: Recommendations for Interventional Trials (SPIRIT) schedule of the trial. The present study is a randomized controlled functional brain imaging trial. One hundred and five functional dyspepsia (FD) patients will be included and randomized equally to one of the three groups: the acupuncture with *deqi* group, the acupuncture without *deqi* group, and the wait-list group. Among 35 patients in each group, 25 participants will be selected randomly to receive fMRI scans. This trial will include a 2-week baseline period, a 4-week treatment period and a 4-week follow-up period. During the 4-week treatment, patients in two acupuncture groups will receive 20 sessions of treatments, puncturing with or without *deqi*, while the wait-list group will not receive acupuncture. Both the outcome assessments and the fMRI scans will be performed at three time points: baseline, end of acupuncture treatments, and the end of follow-up. The central mechanism of the long-lasting effect and the central role of *deqi* on this effect will be analyzed after data collection

This is a parallel-group, randomized controlled study. One hundred and five FD patients will be included and randomized with equal allocation to one of the three groups: acupuncture with *deqi* group, acupuncture without *deqi* group, and the wait-list group. Among 35 patients in each group, 25 participants will be selected randomly to undergo functional magnetic resonance imaging (fMRI) scans. This trial will include a 2-week baseline period, a 4-week treatment period, and a 4-week follow-up period. During the 4-week treatment, patients in two acupuncture groups will receive 20 sessions of treatments puncturing with or without *deqi*, while the wait-list group will not receive acupuncture treatment. Both the outcome assessments and fMRI scans will be performed at three time points including: the baseline, the end of the acupuncture treatments, and the end of follow-up (Figs. 1 and 2). This trial is reported in accordance with the Standard Protocol Items: Recommendations for Intervention Trials (SPIRIT) guidelines [22] (Fig. 2; Additional file 1).

### Sample size

According to our previous study, Nepean Dyspepsia Index (NDI) scores of FD patients decreased by 12.2 points after acupuncture and by 7.8 points after sham acupuncture [23]. In this study, we anticipated a reduction of NDI score by 12 points after acupuncture with the *deqi* sensation and a reduction by 8 points after acupuncture without the *deqi* sensation, and we anticipated a reduction by 4 points in the wait-list control due to regression to the mean effect. With *α* = 0.05, 1 − *β* = 0.8, and a standard deviation of 10, we needed at least 96 participants in total. Considering a dropout rate of 10%, a total of 105 participants will be included in this trial. According to the sample size of previous blood-oxygenation-level-dependent fMRI (BOLD-fMRI) studies, 20 subjects is the reasonable sample size with stable statistical effect [24, 25]. Considering the dropout rate and loss of data due to head motion during fMRI scans, 25 participants from each group will receive fMRI scans in this study.

### Randomization

One hundred and five patients will be randomized into three groups by a third party using simple concealed randomization to avoid possible selection bias. With a computer-generated randomization digital table, patients will be informed using sealed envelopes whether they will receive acupuncture treatment and whether they will receive a fMRI scan. The allocation will be concealed to researchers, outcome assessors, and the statistician.

### Blinding

It is difficult to blind patients as well as acupuncturists for the experience of needle manipulation with or without *deqi*. However, it is feasible to conceal the group assignments from researchers, outcome assessors, and the statistician. Researchers, the acupuncturist, outcome assessors, and the statistician are separate.

### Control

To investigate the influence of the *deqi* sensation on the long-lasting acupuncture effect, we choose acupuncture without *deqi* as the control group. In addition, to avoid the influence of a self-healing tendency, we design a wait-list group in which FD patients will not receive acupuncture treatment.

### Participants and recruitment

Based on the Rome III criteria and current academic literature, FD comprises three subtypes: postprandial distress syndrome (PDS), epigastric pain syndrome (EPS), and a subtype with overlapping PDS and EPS features [26]. Among the three subtypes, FD patients diagnosed with PDS are most common in the epidemiology [27], and no significant differences were found in the acupuncture response between FD patients in PDS and FD patients in EPS in our previous study [28]. Therefore, FD patients with PDS, or the subtype with overlapping PDS and EPS features but PDS dominating will be recruited if they match the inclusion criteria. They will be briefly introduced to this study and informed about the benefits as well as possible risks associated with needling (bleeding, hematoma, or fainting). Then, the participants will be required to fill in consent forms and are free to withdraw from the study at any time without any specific reason, penalty or loss of benefits.

All researchers responsible for screening patients will be trained using the Rome III criteria in the pretrial period. They will screen a group of participants and then compare and fix their screening results to achieve homogeneity.

### Inclusion criteria

Participants who meet each of the following criteria will be included: (1) matching the Rome III criteria on FD with subtype PDS or subtype with overlapping PDS and EPS features but PDS dominating; (2) aged between 18 and 45 years, and right handed; (3) without intake of any gastroenteric dynamic drugs in 2 weeks before enrollment; (4) not participating in any other clinical trials; and (5) having signed a written informed consent form.

### Exclusion criteria

Participants who meet one of the following criteria will be excluded: (1) FD with subtype EPS or subtype with overlapping PDS and EPS features but EPS dominating; (2) having esophagitis, gastric atrophy, or erosive gastroduodenal lesions on endoscopy, cholecystitis, gallstones; (3) having psychological problems, being unconscious, having a history of mental disorders, being unable to cooperate in outcome assessment; (4) having aggravating malignant tumors or other serious consumptive diseases, infectious, bleeding diseases, etc.; (5) having used aspirin, steroids, nonsteroidal anti-inflammatory drugs, selective serotonin-reuptake inhibitors, medication affecting gastrointestinal motility, or other drugs; (6) pregnant women, or intent to, or in, the breast-feeding period within 6 months; (7) having any contraindications to fMRI scan, such as having a cardiac pacemaker, defibrillator, metal stents, or electronic device in the body, having an intraocular metal foreign body, having claustrophobia, hyperpyrexia, etc.

### Recruitment strategies

All patients will be recruited from outpatients and inpatients of the Digestive Department of the First Teaching Hospital of Chengdu University of Traditional Chinese Medicine (TCM) and the campus of Chengdu University of TCM between January 2016 to December 2018. Patients interested in this trial will be first evaluated by physicians. If they meet the criteria and decide to participate, they will be asked to sign the informed consent. Then, they will be included in this trial for randomization.

### Interventions

**Fig. 3:**
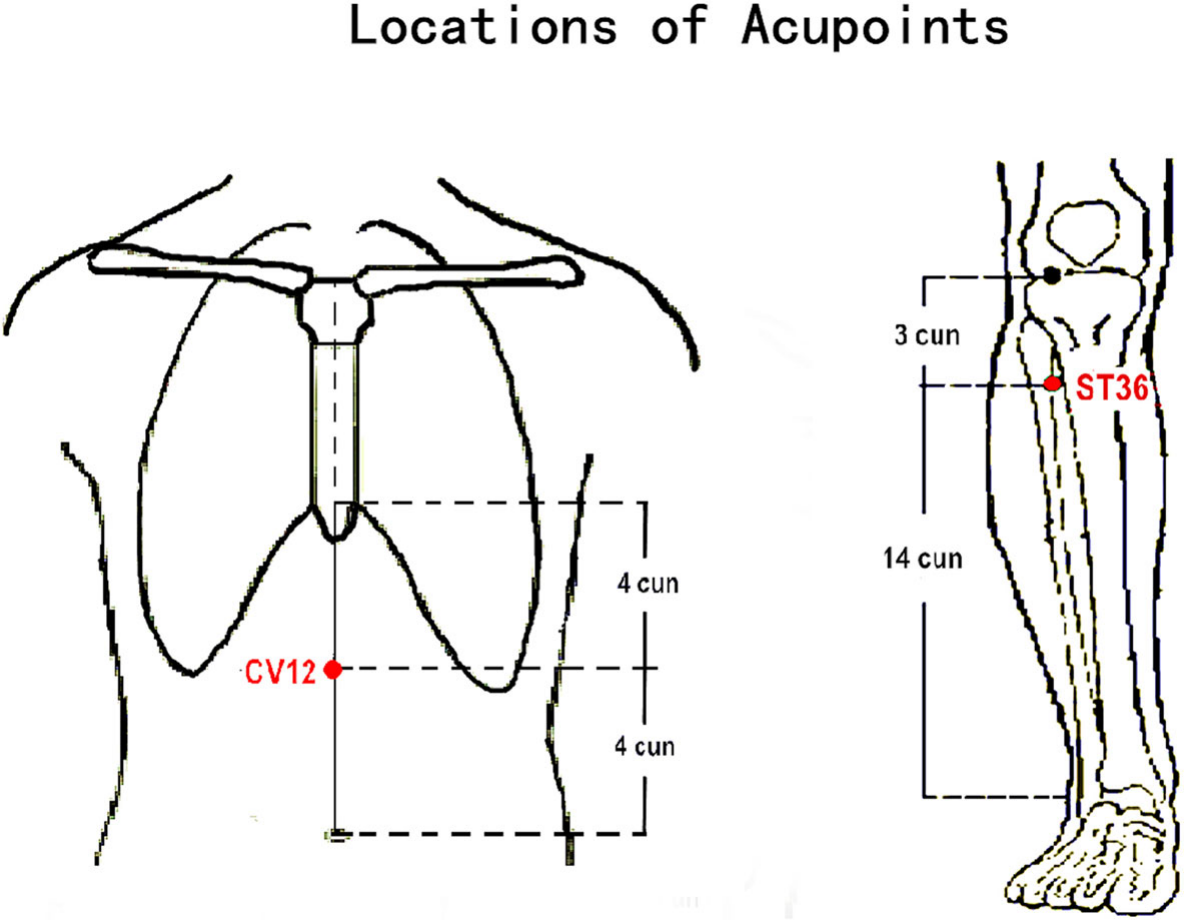
Locations of acupoints. Locations of acupoints: ST36 (*Zusanli*), on the anterior lateral side of the shank, 3 *cun* below ST35 (*Dubi*), one horizontally placed finger distance lateral to the anterior border of the tibia. CV12 (*Zhongwan*), on the anterior median line of the upper abdomen, 4 *cun* above the navel. These two acupoints have been used in our previous study by Shuai Yin et al. (Cerebral mechanism of puncturing at *He-Mu* point combination for functional dyspepsia: study protocol for a randomized controlled parallel trial. *Neural Regen Res.* 2017;12: 831–840.), so as to provide a stable acupuncture effect

The acupoints used in the study include unilateral *Zhongwan* (CV12) and bilateral *Zusanli* (ST36) (Fig. 3). The combination of CV12 and ST36 is commonly used for treating FD, and has been used in our previous studies [29].

Acupuncture treatment will be performed by two licensed acupuncturists with clinical experience over 3 years. The acupuncturists will be trained and take an examination before participating in this trial. The two acupuncture groups will receive 20 sessions’ treatment over a period of 4 weeks. The acupuncture treatment is performed once per day for 5 days continuously then at 2-day interval.

#### Acupuncture with the *deqi* group

After the skin has been cleaned with tincture iodine and alcohol, the sterile filiform needles (0.25 mm in diameter, 40 mm in length, Hwatuo, Suzhou, China) are inserted perpendicularly into the points for 21–26 mm. Then, the needle is twisted between 90 and 180°, lifted and thrusted in an even amplitude between 0.3 cm to 0.5 cm, 60 times to 90 times per minute. After the *deqi* response (including soreness, numbness, distention, and heaviness) is obtained, the needle will be retained in the skin for 30 min, still being manipulated every 10 min twice for 10 to 15 s.

#### Acupuncture without the *deqi* group

To avoid the *deqi* response, no needle manipulation will be performed after the perpendicular insertion.

#### Wait-list group

Patients will not receive acupuncture treatment during the study. For *deqi* it is important in achieving clinical efficacy that these patients will be treated by acupuncture with *deqi* after finishing the study.

Participants with pain, bleeding, fainting, or other severe discomforts should be discontinued from acupuncture treatment and processed immediately. During the acupuncture treatment and follow-up, all participants are usually not allowed to use FD symptomatic or emotional relief medication. But if required, participants can be permitted to receive acute stomach disorder treatment, and the type and dosage of medication used should be recorded in the case report form (CRF).

### fMRI scan

Brain images will be acquired on a 3.0-T magnetic resonance scanner (Siemens, Munich, Germany) at Huaxi Magnetic Resonance Research Center, West China Hospital of Sichuan University, Chengdu, China. The scan will be performed at three time points including: baseline, the end of the treatment, and the follow-up. During the scan, each participant is blindfold, with their ears plugged, and is required to undergo a high-resolution, three-dimensional, T1-weighted and BOLD-fMRI sequence. Three-dimensional anatomic image parameters are as follows: repetition time/echo time = 1900 ms/2.26 ms, slices = 30; matrix size =128 × 128, field of view = 256 × 256 mm^2^, slice thickness = 1 mm. The BOLD images will be acquired as: repetition time/echo time = 2000 ms/30 ms, flip angle = 90°, slices = 30; matrix size = 128 × 128 mm^2^, field of view = 240 × 240, slice thickness = 5 mm.

### Patient safety

To provide health evaluation before randomization, physical examination for participants will be conducted including gastroscope examination, abdominal ultrasound, blood biochemical test (ALT, AST, BUN, serum creatinine), and electrocardiogram examination. To assess the risks associated with acupuncture, the blood biochemical test will be completed again at the end of treatment. Adverse events, such as pain, bleeding, fainting, or other severe events, should be processed immediately and recorded in the CRF carefully.

### Outcome assessments

The following outcomes will be assessed by independent outcome assessors. These assessors are trained before participating in this trial and blinded to the randomization. Outcome data for participants either completed or discontinued during the study will be collected and recorded.

Clinical outcome assessments include: symptoms, QOL, and psychosocial state. All evaluations will be performed at three time points (the baseline, the end of 20 sessions of acupuncture and the end of follow-up, respectively) (Fig. 2).

The Nepean Dyspepsia Index (NDI) is primarily used to measure the symptoms and QOL for FD patients [30]. It includes the following two parts. The symptom checklist measures 15 upper gastrointestinal symptoms regarding frequency, intensity, and level of discomfort. The QOL checklist includes four aspects, namely life interference (13 items), knowledge/control (seven items), eating/drinking (three items), and sleep/disturbance (two items). Higher scores indicate more severe symptoms and poorer QOL. Our prior research found that the Chinese version of the NDI was reliable and valid for measuring QOL in Chinese FD patients [31].

The short form Leeds Dyspepsia Questionnaire (SF-LDQ) [32] is also performed to assess the symptom severity. The SF-LDQ is a validated and reliable tool with five questions instrument to measure the patients’ dyspeptic symptoms. Five questions are used for five symptoms including epigastric pain, postprandial distention, indigestion, heart-burn, and nausea. By using a five-point Likert scale, the symptoms are graded for severity from very mild to very severe: no symptoms (0 point), very mild symptoms without influence of regular work (1 point), mild symptoms with influence of regular work (2 points), moderate symptoms (3 points), severe symptoms (4 points), and extremely severe symptoms (5 points). The severity of dyspepsia is evaluated by total scores on these five symptoms. Higher total scores indicate more severe symptoms.

As emotional changes, such as anxiety and depression, are commonly seen accompanying symptoms in FD patient, the Zung Self-Rating Anxiety Scale (SAS) [33], the Zung Self-Rating Depression Scale (SDS) [34], the Beck Anxiety Inventory (BAI) [35], and the Beck Depression Inventory (BDI) [36] are used in this study to evaluate the psychological condition of patients.

### Needle sensation evaluations

Participants in the two acupuncture groups will be asked to fill the needle sensation evaluation form after receiving each acupuncture treatment. The form is based on the Chinese version of modified Massachusetts General Hospital Acupuncture Sensation Scale (C-MASS) [37, 38], and its validity and reliability have been examined [38, 39]. In the form, the Visual Analog Scale (VAS) [40] is selected to quantitatively evaluate the *deqi* sensation after each acupuncture treatment. This is a 10-point scale with anchor words “none” (no score), “mild” (about two scores), “moderate” (around five scores), “severe” (almost eight scores), and “unbearable” (nearly ten scores) spaced along the continuum. The scale will be used to access evaluate the *deqi* sensations (including soreness, numbness, fullness, heaviness, aching), respectively. Moreover, if the scale does not completely describe the needle sensations that subjects experienced, subjects can add what they feel in a blank row below the scale.

Since some participants were sensitive while some were not, both acupuncture groups’ participants will be re-divided into the “actual *deqi* group” and “actual without *deqi* group” according to the median of the mean needle sensation score across 20 times treatment. Then the “actual *deqi* group” and “actual without *deqi* group” will be compared with both clinical data and fMRI data.

### Data management and monitoring

The CRFs for each participant will be prepared in both paper and electronic versions. Only outcome assessors have access to CRFs and will perform the double-data entry. The Evidence-based Medicine Center of the university will be responsible for monitoring the study as well as the data every 3 months.

## Data analysis

### Clinical data analysis

The intention-to-treat analysis will be used for all allocated participants in the baseline. This study focuses on the lasting-effect of acupuncture, and the observed data will be used as the primary analysis and last observation-carried-forward (LOCF) analysis will be taken as a sensitivity analysis. Clinical outcome will be analyzed using SPSS 22.0 statistics software (IBM SPSS Statistics, IBM Corp, Somers, NY, USA). Data on Skewed distribution will be normalized. Means, standard deviations, and 95% confidence intervals (CIs) will be used to describe the continuous data.

The demographic and baseline characteristics among three groups will be compared using one-way analysis of variance (ANOVA). A *χ*^2^ test will be used for categorical variables. Due to the occurrence of self-healing tendency in FD, it is important to testify the existence of acupuncture effect. The clinical outcome NDI and SF-LDQ scores will be compared between the acupuncture group and the wait-list group among three time points by using the repeated measures ANOVA (two independent groups with repeated measures). Then, the acupuncture group with and without *deqi* at three time points will be primarily compared using repeated measures model.

A two-sided test will be used during the analysis, with a significance level of 0.05. The data will be analyzed by statisticians who are blinded to the test settings.

### fMRI data analysis

The fMRI scan data will be preprocessed and analyzed by SPM12 software (SPM12, Wellcome Department of Imaging Neuroscience, London, UK; http://www.fil.ion.ucl.ac.uk/spm/) performing on MATLAB 8.6 (Mathworks, Inc., Natick, MA, USA). After data preprocessing, regional homogeneity (ReHo), amplitude of low-frequency fluctuation (ALFF) and functional connectivity (FC) will be used to investigate the different cerebral responses between *deqi* and without *deqi*. Then correlation analysis will be conducted between clinical data and fMRI image data.

## Discussion

Based on ancient records and recent RCT results, acupuncture has a long-lasting effect for treating dyspepsia, but its central mechanism and influential factors remain uncertain. Considering the importance of the *deqi* sensation for the acupuncture effect, we have designed a randomized controlled fMRI trial to explore the central mechanism of the acupuncture long-lasting effect and the influence of the *deqi* sensation on the long-lasting effect for the first time.

### *Deqi* may play an essential role in achieving the long-lasting effect for its definite influence on acupuncture effect

The long-lasting effect, as one of the most commonly phenomenon and important feature of acupuncture effect, refers to the perception of a curative effect for a period of time after termination of acupuncture treatment. It has been recorded in a number of ancient Chinese medical books and reported in many clinical trials such as acupuncture treatment for migraine [7], FD [4], cervical spondylosis [41], chronic pain in the knee joints [42], and others [5, 6]. For example, in a systematic review including 13 studies on chronic pain in knee joints, three of them found that the acupuncture efficacy still remained in the following 6 to 12 months after treatment [42]. Our previous multicenter clinical trials on acupuncture treatment for FD also demonstrated that the effects of puncturing on acupoints were not only significantly different from those on non-acupoints at the end of 4 weeks’ treatment, but also in the 4 weeks and 12 weeks after the termination of treatment [4]. All the above indicated the existence of the long-lasting effect and its importance in keeping acupuncture efficacy.

*Deqi,* also called the arrival of *qi*, or the needle sensation, is a series of multidimensional and intense sensations including numbness, soreness, distention, heaviness, and dull pain, etc. that patients experience during acupuncture stimulation [43]. A great amount of ancient acupuncture classics recorded that “ the achievement of acupuncture effect depends on the *qi* arrival” (“*Huangdi Neijing*,” *The Inner Canon of Huangdi*), and “the quicker the *qi* arrives, the faster the acupuncture effect acquires” (“*Biao You Fu*,,” *a Poetic Prose on the Elucidation of Acupuncture*). Modern clinical trials have considered that *deqi* plays an essential role in the process of achieving the acupuncture effect [37, 44–47]. For example, a study on Bell’s palsy found that acupuncture with *deqi* can induce a greater therapeutic effect, and stronger intensity of *deqi* was associated with better therapeutic effects [47].

Considering the definite influence of *deqi* on acupuncture effect, we noted that *deqi* may play a vital role in achieving the long-lasting effect. Our previous study on migraine also demonstrated that the therapeutic effect induced by acupuncture with *deqi* can last for 20 weeks after 4 weeks’ treatment [48]*.* The results not only confirmed the existence of the long-lasting effect, but also presented its intimate relationship with *deqi*. So, this study tries to firstly re-identify the influence of *deqi* on the long-lasting effect, and then further explore the mechanism of how *deqi* plays the central role in the long-lasting effect.

### The potential central mechanism of the long-lasting effect has not been well investigated

Although the long-lasting effect has been reported by a number of RCTs [9, 10, 42, 49] and animal studies [50, 51], most of them focused on identifying the existence of the long-lasting effect. An animal study on irritable bowel syndrome (IBS) reported that the P2X3 receptor, which played an important role in visceral pain, was downregulated at the sixth week after the electroacupuncture (EA) treatment, suggesting that the regulation of the P2X3 receptor may be associated with the mechanism underlying the production of the long-lasting effect [51].

Since central integration is the key for achieving acupuncture effect, using functional brain imaging techniques including fMRI, positron emission tomography (PET) / computed tomography(CT), electroencephalography (EEG), and magnetoencephalography (MEG) to explore the central mechanism of acupuncture effect has attracted increasing attention worldwide in the last 20 years. In our previous academic literature study, we found that in total 168 papers on acupuncture functional brain imaging were published from 1995 to 2014 [52]. These studies mapped the brain regions involving in acupuncture effect. For example, Hui. and her co-investigators found that acupuncture with *deqi* can elicit extensive negative BOLD signal changes (deactivation) in the limbic-paralimbic-neocortical network (LPNN) and positive BOLD signal changes in somatosensory regions of the brain [53]. This study provided functional brain imaging evidence of the influence of *deqi* on cerebral activity.

However, little work has been done in revealing the potential central mechanism of the long-lasting effect and how *deqi* influences the production of the long-lasting effect, which is worthy of investigation. Therefore, this trial aims to investigate the central mechanism of the acupuncture long-lasting effect and the central influence of *deqi* based on re-identifying the existence of the long-lasting effect on acupuncture for FD.

### The quality control program is the precondition for the result reliability

To improve the result reliability of this study, we design the quality control program from the following four aspects: (1) patient selection for the influence of age and handedness on cerebral function and structure have long been investigated, this study restricts the subjects to age between 18 to 45 years and being right-handed for baseline homogeneity; (2) sample size to obtain stable statistical power; we include 35 patients in each group for clinical evaluation and 25 patients in each group for the central mechanism study; and (3) fMRI scan; all scans are performed in the morning with the same scanner and operator. Scans on female participants are performed during the same menstrual cycle. Moreover, during the 24 h before scanning, participants will be asked to maintain their regular lifestyle and avoid overexertion and staying up late. No permission with alcohol, tobacco, tea, and coffee. Before being scanned, the emotional state of each participant will be evaluated via the emotional state assessment scales. To exclude significant emotional changes, during the scan participants will be asked to close their eyes and use a blindfold and to plug their ears with earplugs, stay awake, and not to speak. The scan room keeps the temperature between 18 and 22 °C, with humidity greater than 60% and noise less than 150 dB; (4) acupuncture manipulation; the acupuncture treatment will be performed by two experienced acupuncturists using a standard operation procedure. The *Deqi* sensation will be evaluated and recorded with the VAS after each acupuncture treatment.

In conclusion, the long-lasting effect is an important feature in acupuncture efficacy, and *deqi* may be one vital factor in producing the long-term effect. This study is the first functional brain imaging study which focuses on exploring the central mechanism of the long-lasting effect and the central influence of *deqi* on this effect with strict quality control. The results may provide a new approach to investigate the mechanism of the long-lasting effect.

### Trial status

This trial was registered in June 2015 (Registration Number: ChiCTR-IOR-15006523, the protocol version number: F2.0). Due to the funding delay, recruitment, preparation, and other reasons, this trial started at the beginning of 2016. Its deadline would correspondingly be postponed to December 2018. The first participant was included on 18 May 2016. To date, 61 participants have been recruited. This trial is still recruiting patients. The recruitment will be completed by approximately 30 November 2018.

## Additional file

Additional file 1: Standard Protocol Items: Recommendations for Interventional Trials (SPIRIT) 2013 Checklist: recommended items to address in a clinical trial protocol and related documents*. (DOC 127 kb)

## Abbreviations

ALFF: Amplitude of low-frequency fluctuation
BAI: Beck Anxiety Inventory
BDI: Beck Depression Inventory
BOLD-fMRI: Blood oxygenation level-dependent fMRI
EA: Electroacupuncture
EEG: Electroencephalography
FC: Functional connectivity
FD: Functional dyspepsia
FGID: Functional gastrointestinal disorders
fMRI: Functional magnetic resonance imaging
IBS: Irritable bowel syndrome
LDQ: Leeds Dyspepsia Questionnaire
LPNN: Limbic-paralimbic-neocortical network
MEG: Magnetoencephalography
NDI: Nepean Dyspepsia Index
PET/CT: Positron emission tomography/computed tomography
PPI: Proton pump inhibitor
QOL: Quality of life
RCT: Randomized clinical trial
ReHo: Regional homogeneity
SAS: Zung Self-Rating Anxiety Scale
SDS: Zung Self-Rating Depression Scale
SPIRIT: Standard Protocol Items: Recommendations for Intervention Trials
TCM: Traditional Chinese Medicine
VAS: Visual Analog Scale
